# The Acquisition of a Memory Phenotype by Murine CD4^+^ T Cells Is Accompanied by a Loss in Their Capacity to Increase Intracellular Calcium

**DOI:** 10.1155/1992/31573

**Published:** 1992

**Authors:** Lex Nagelkerken, Anita Hertogh-Huijbregts

**Affiliations:** Section of Immunology TNO Institute of Aging and Vascular Research TNO P.O. Box 430 Leiden 2300 AK The Netherlands

**Keywords:** CD4+, naive, memory, calcium, aging

## Abstract

During the process of aging, the fraction of CD4^+^ T Cells with a naive phenotype, that is, Pgp-1^-^ CD45RB^High^MEL-14^+^, decreases in favor of CD4^+^ T memory cells. Total CD4^+^ T cells from aged mice displayed a diminished calcium response to anti-CD3 and even ionomycin as compared to the cells from young mice, and this was related to the changed composition of the CD4^+^ T-cell population. Regardless the age of the donor mice, naive CD4^+^ T cells effectively increased intracellular calcium, whereas memory CD4^+^ T cells were impaired in this regard. In addition, a heterogeneity in the differentiation stage of the naive CD4^+^ T cells was shown by the observation that calcium mobilization in naive CD4^+^ T cells from young mice was more profound than that in their aged counterparts. These data thus indicate that during the acquisition of a memory phenotype, murine CD4^+^ T cells lose the capacity to increase intracellular calcium, which in turn may be responsible for the decreased level of IL-2 production by these cells.


*Developmental Immunology*, 1992, Vol. 3, pp. 25–34
Reprints available directly from the publisher
Photocopying permitted by license only

© 1992 Harwood Academic Publishers GmbH
Printed in the United States of America

### The Acquisition of a Memory Phenotype by Murine $CD4^+$ T Cells is Accompanied by a Loss in Their Capacity to Increase Intracellular Calcium

LEX NAGELKERKEN* and ANITA HERTOGH-HUIJBREGTS

*Section of Immunology, TNO Institute of Aging and Vascular Research TNO, P.O. Box 430, 2300 AK Leiden, The Netherlands*

During the process of aging, the fraction of $CD4^+$ T cells with a naive phenotype, that is, $Pgp\text{-}1^-$ $CD45RB^{High}MEL\text{-}14^+$, decreases in favor of $CD4^+$ T memory cells. Total $CD4^+$ T cells from aged mice displayed a diminished calcium response to anti-CD3 and even ionomycin as compared to the cells from young mice, and this was related to the changed composition of the $CD4^+$ T-cell population. Regardless the age of the donor mice, naive $CD4^+$ T cells effectively increased intracellular calcium, whereas memory $CD4^+$ T cells were impaired in this regard. In addition, a heterogeneity in the differentiation stage of the naive $CD4^+$ T cells was shown by the observation that calcium mobilization in naive $CD4^+$ T cells from young mice was more profound than that in their aged counterparts. These data thus indicate that during the acquisition of a memory phenotype, murine $CD4^+$ T cells lose the capacity to increase intracellular calcium, which in turn may be responsible for the decreased level of IL-2 production by these cells.

KEYWORDS: $CD4^+$ T cells, naive, memory, calcium, aging.

### INTRODUCTION

Aging is accompanied by a decrease in the capacity of $CD4^+$ T cells to produce interleukin-2 (IL-2; Gillis et al., 1981; Thoman and Weigle, 1981). However, it still remains to be established to what extent this phenomenon is due to intrinsic defects related to the process of aging or to a different stage of differentiation of the $CD4^+$ T cells. The expression of the IL-2 gene is dependent on the induction of the nuclear factor of activated T cells (NFAT-1; Shaw et al., 1988; Crabtree, 1989), which in turn is regulated by at least two intracellular events that are the result of perturbation of the CD3-TCR complex, that is, an increase in intracellular calcium and the activation of protein kinase C (Verweij et al., 1990; Hivroz-Burgaud et al., 1991). Interestingly, it has been shown that T cells from aged mice (Miller et al., 1987) and man (Grossmann et al., 1989) are impaired with regard to their ability to mobilize cytoplasmic calcium and this might be one of the reasons for a lower level of IL-2 production. However, several groups, including us, have recently demonstrated that the differentiation stage of the $CD4^+$ T-cell population changes during the aging process (Lerner et al., 1989; Ernst et al., 1990; Nagelkerken et al., 1991; Dobber et al., 1992), pointing to a decrease in the number of naive $CD4^+$ T cells in favor of memory cells. In the mouse, we have demonstrated that this increase in the fraction of $CD4^+$ T memory cells is functionally related to the decrease in the production of IL-2 and an increase in the production of IL-4 and interferon-$\gamma$ (Nagelkerken et al., 1991). From this and other studies (Bottomly et al., 1989; Lee et al., 1990), it appeared that following polyclonal stimulation, naive $CD4^+$ T cells were the main producers of IL-2. Therefore, an age-related decrease in IL-2 production is not necessarily a consequence of an intrinsic defect, but may rather reflect functional differences between naive and memory cells. Likewise, also, the ability of T cells to mobilize calcium may be dependent on the differentiation stage of these

*Corresponding author.

cells. In the present study, we addressed this possibility by studying calcium mobilization in CD4$^{+}$ T-cell subsets from mice of different ages.

### RESULTS

### Phenotype of CD4$^{+}$ T Cells and CD8$^{+}$ T Cells in Young and Aged Mice

Major phenotypic changes occur within the CD4$^{+}$ T-cell population during the process of aging. This has previously been demonstrated on the basis of an increased expression of Pgp-1 and a decreased expression of CD45RB (Lerner et al., 1989; Ernst et al., 1990; Nagelkerken et al., 1991). In Fig. 1, it is shown that these two markers are more or less reciprocal by performing triple staining experiments on unseparated spleen cells from mice of different ages. At an age of 3 months, the majority of the CD45RB$^{++}$CD4$^{+}$ T cells from CBA/Rij mice does not yet express Pgp-1, and particularly the proportion of this subset decreases during the first year of life, probably as a consequence of thymic involution. This decrease is accompanied by an increase in the fraction of CD45RB$^{++}$Pgp-1$^{+}$CD4$^{+}$ T cells. In addition, the number of Pgp-1$^{++}$CD4$^{+}$ T cells increases during aging. Although the majority of these cells are CD45RB$^{-}$, a substantial fraction of the cells expresses CD45RB in low density and this is most evident in 15-month-old mice. Below, CD45RB$^{-}$ and CD45RB$^{+}$ will collectively be indicated as CD45RB$^{Low}$ cells; CD45RB$^{++}$ cells will be indicated as CD45RB$^{High}$ cells. Based on functional studies performed by other investigators (Bottomly et al., 1989; Lee et al., 1990), these phenotypic changes should be interpreted as a decrease in the fraction of naive cells in favor of memory cells. This conclusion is further supported by our observation that the fraction of MEL-14$^{+}$CD4$^{+}$ T cells decreased during aging, because this marker also has been recently associated with memory cells (Bradley et al., 1992).

As can be concluded from Fig. 2, about 90% of young CD4$^{+}$ T cells are MEL-14 as compared to about 35% in 2-year-old mice. By contrast, only a slight decrease was noted in the fraction of MEL-14$^{+}$CD8$^{+}$ T cells, although Pgp-1 expression was also increased on old CD8$^{+}$ T cells (data not shown).

### Age-Related Decline in Calcium Mobilization in T Cells

As shown before, the stage of differentiation on the T cells is related to the age of the donor mice. To study the influence of differentiation on the ability to increase cytoplasmic calcium, we purified CD4$^{+}$ T cells from mice of different ages, labeled them with INDO-1, and studied their response to anti-CD3 antibodies or to ionomycin. By employing 2C11 as the stimulating anti-CD3 antibody, cross-linkage was required to induce calcium mobilization. This was achieved employing a goat-anti-rat-Ig cross-reactive with hamster IgG. A typical experiment is shown in

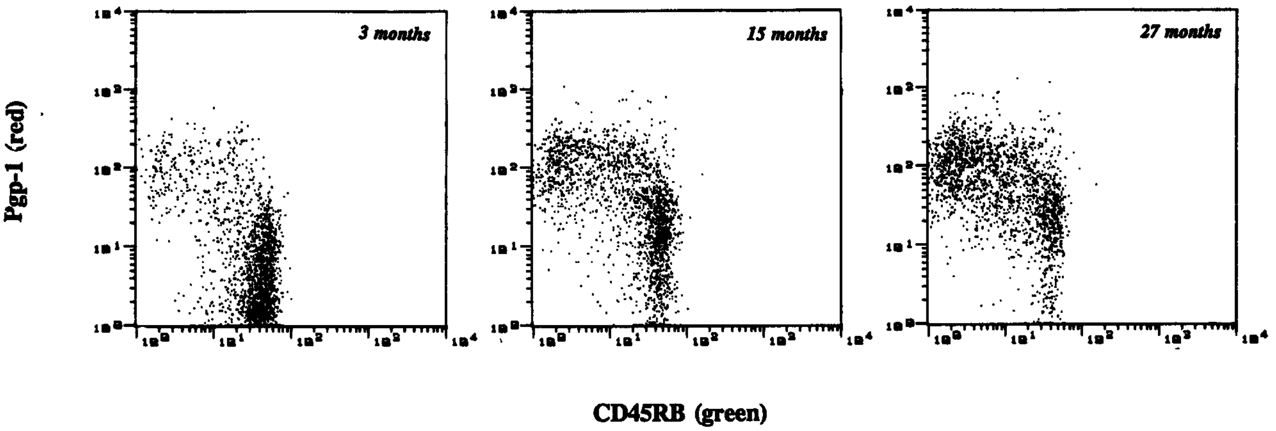

FIGURE 1. Age-related changes in the composition of the CD4$^{+}$ T-cell population. For each age group, spleen cells from four mice were pooled and analyzed for the simultaneous expression of CD4, CD45RB, and Pgp-1. Only gated CD4$^{+}$ T cells are shown.

Fig. 3A. It can be seen that the addition of this second antibody resulted in an calcium flux in about 60% of the CD4⁺ T cells derived from 3-month-old mice. When CD4⁺ T cells were derived from 27-month-old mice, a substantially lower fraction of the cells (about 25%) responded to anti-CD3. Moreover, the response by cells from aged mice was heterogeneous in that increasing numbers of individual cells responded less vigorously (data not shown). Results obtained with CD4⁺ T cells from 15-month-old mice were similar to those obtained with cells from 27-month-old mice, indicating that the decline in calcium mobilization is not likely to be due to age-related defects (data not shown). To establish whether the less vigorous response to anti-CD3 was due to the lower level of expression of CD3 that we found previously with T cells from aged mice (Hertogh-Huijbregts et al., 1990), cells were also stimulated with ionomycin. Under these conditions, the fraction of responding cells increased in young and aged mice, although differences between cells from young and aged mice (90% and 65% responding cells, respectively) remained (Fig. 3A). Similar results were obtained with CD8⁺ T cells (data not shown).

To examine whether the decrease in the ability to mobilize calcium was a general phenomenon in aging animals, we also studied calcium mobilization in splenic B cells. As shown in Fig. 3B, no differences were found between B cells from young or aged mice, regardless whether the cells were stimulated with anti-Ig antibodies or with ionomycin.

### Calcium Mobilization in Subpopulations of CD4⁺ T Cells

In order to establish whether the age-related decline in calcium mobilization was due to the changes in the composition of the CD4⁺ T-cell population, we next studied calcium mobilization in CD4⁺ T-cell subsets from mice of different ages. This was done in triple staining experiment: spleen cells were labeled with Fluo-3, and stained with the combination of anti-$CD4^{Red613}$ and Phycoerythrin-conjugated anti-Pgp-1, which allowed us to study the response of Pgp-1⁻, Pgp-1⁺, and Pgp-1⁺⁺CD4⁺ T cells simultaneously. In a comparable way, we studied $CD45RB^{High}$ and $CD45RB^{Low}$ CD4⁺ T cells. At the concentrations of

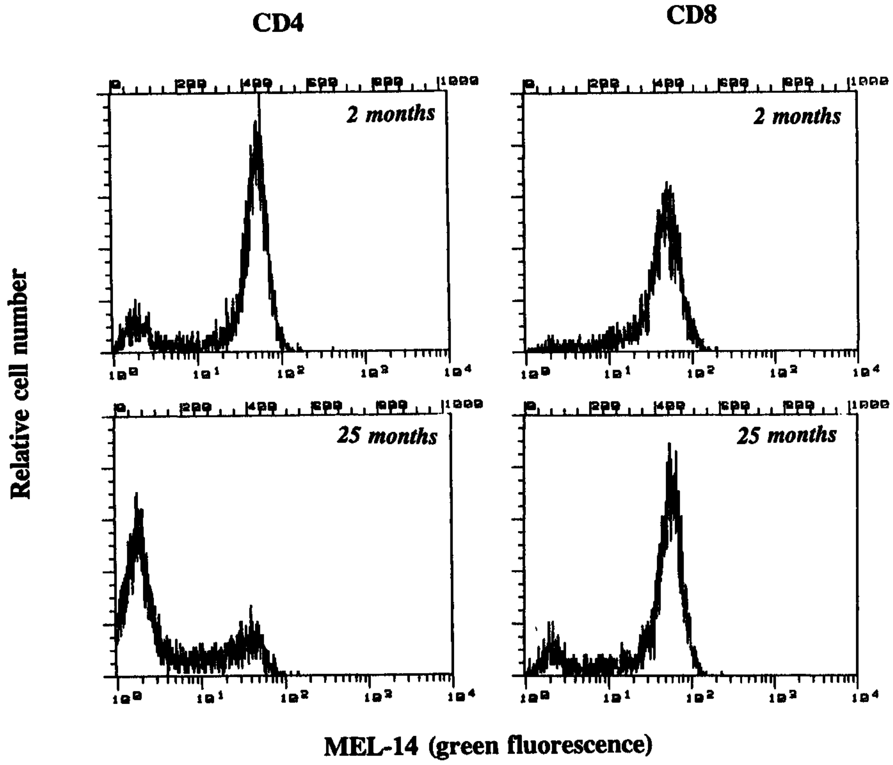

FIGURE 2. Age-related decline in the fraction of splenic MEL-14⁺CD4⁺ T cells, but not in the fraction of MEL-14⁺CD8⁺ T cells. MEL-14 density (green fluorescence) on gated CD4⁺ T cells or CD8⁺ T cells is displayed.

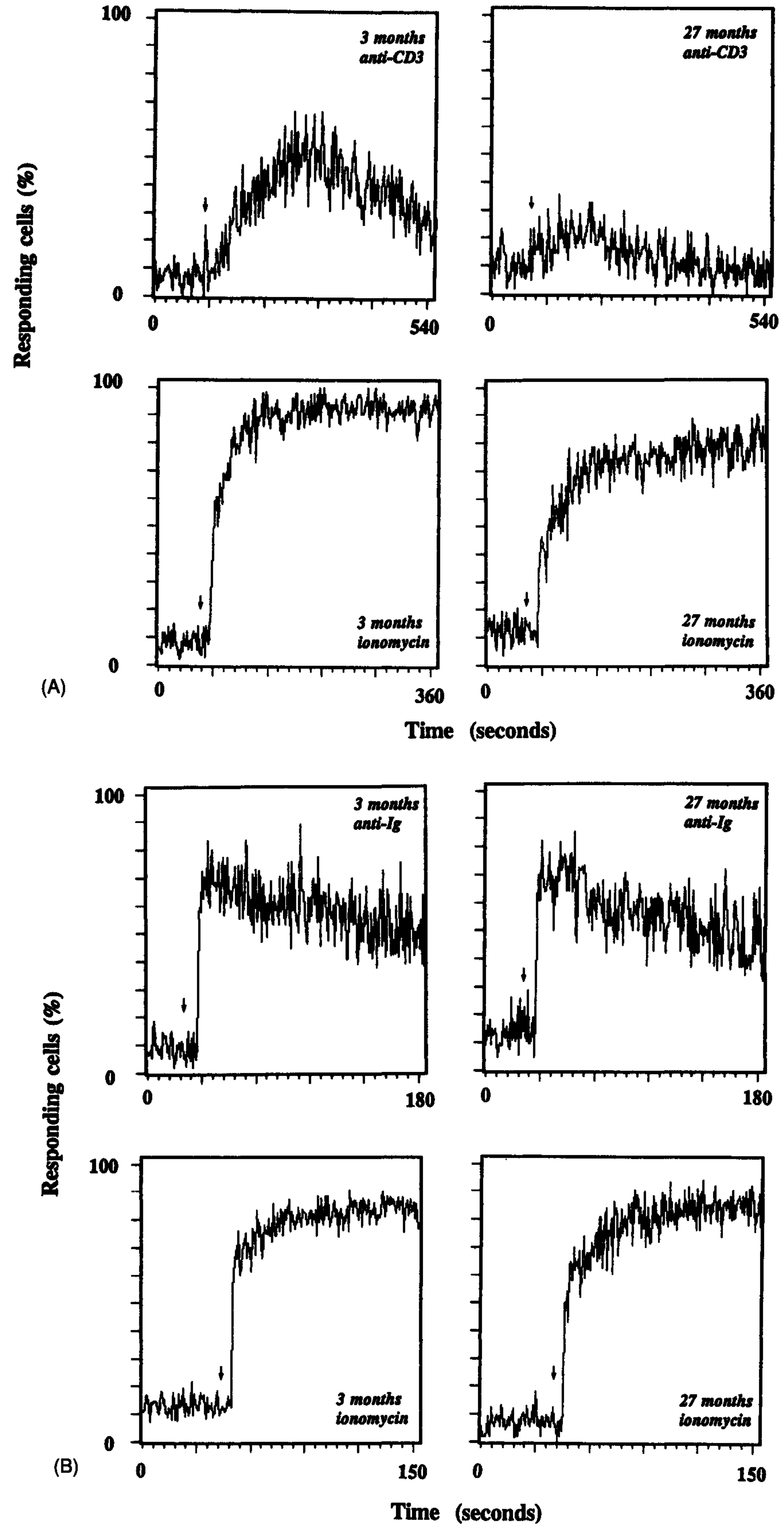

FIGURE 3. Calcium mobilization is impaired in CD4+ T cells, but not in B cells from aged mice. CD4+ T cells (A) or B cells (B) were isolated from a pool of spleen cells from four CBA/Rij mice of the indicated age, labeled with INDO-1 and stimulated (indicated by arrow) with anti-CD3, anti-Ig, or ionomycin. The results shown for the CD4+ T cells and the B cells are representative for five experiments and three experiments, respectively.

antibody we employed, anti-Pgp-1 did not affect the calcium response, regardless of whether the cells were stimulated by anti-CD3 or ionomycin. By contrast, anti-CD45RB inhibited the calcium response to anti-CD3 but not to ionomycin. The results are expressed as histograms of fluorescence intensity before stimulation (base level) and immediately after the addition of the stimulus at the peak of the response.

A typical experiment is shown in Fig. 4. After stimulation with ionomycin, it is observed that regardless of the age of the mice, Pgp-1$^{++}$CD4$^{+}$ T cells display only a slight increase in fluorescence intensity, whereas Pgp-1$^{+}$ cells respond more adequately and the highest response is noted in Pgp-1$^{-}$ cells. A quantitative representation of this experiment is given in Table 1, where the increase in mean fluorescence intensity after stimulation is depicted. It can be seen that if cells from mice of different ages are compared also an age-related decrease in responsiveness can be noted, regardless of the expression level of Pgp-1. Similar conclusions can be drawn, on the basis of stimulation with anti-CD3, although then only

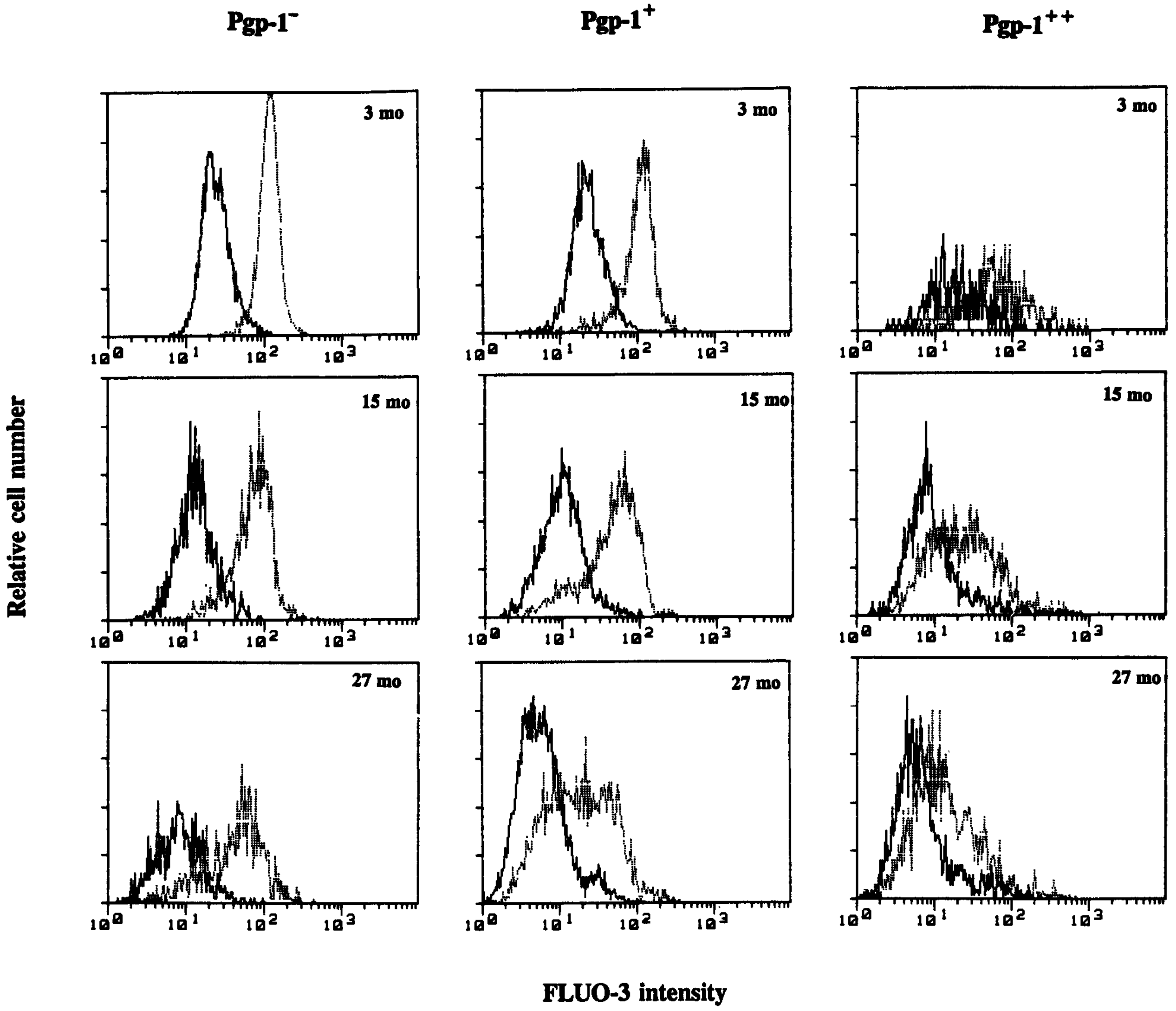

FIGURE 4. Relation between Pgp-1 expression and calcium mobilization. FLUO-3-labeled spleen cells from mice of different ages stained for CD4 and Pgp-1 were stimulated with ionomycin. Histograms of FLUO-3 intensity before stimulation (thick line) and at the peak of the response (thin line) are displayed. Results representative for three experiments are shown.

marginal responses were obtained with Pgp-1$^{++}$ cells. Cells that had been incubated with anti-CD45RB displayed a subdued calcium response to anti-CD3, although it could be still be concluded that CD45RB$^{High}$ cells responded better than CD45RB$^{Low}$ cells (Table 1). Ionomycin bypassed this inhibitory effect. As shown in Fig. 5 and Table 1, CD4$^{+}$ T cells defined on the basis of a high CD45RB expression responded better to ionomycin than CD45RB$^{Low}$ CD4$^{+}$ T cells, even

TABLE 1
A Decline in Calcium Mobilization Accompanies the Differentiation of CD4$^{+}$ T Cells

| Age (months) | Stimulus | Increase in FLUO-3 intensity[a] | | | | |
|---|---|---|---|---|---|---|
| | | Pgp-1$^{-}$ | Pgp-1$^{+}$ | Pgp-1$^{++}$ | CD45RB$^{High}$ | CD45RB$^{Low}$ |
| 3 | anti-CD3 | 68 | 50 | 11 | 30 | 15 |
| 3 | ionomycin | 95 | 86 | 66 | 108 | 78 |
| 15 | anti-CD3 | 44 | 22 | 6 | 14 | 8 |
| 15 | ionomycin | 67 | 43 | 28 | 63 | 33 |
| 27 | anti-CD3 | 38 | 17 | 4 | 12 | 3 |
| 27 | ionomycin | 46 | 21 | 14 | 35 | 15 |

[a]Mean fluorescence intensity at the peak of the response—mean fluorescence intensity before stimulation.

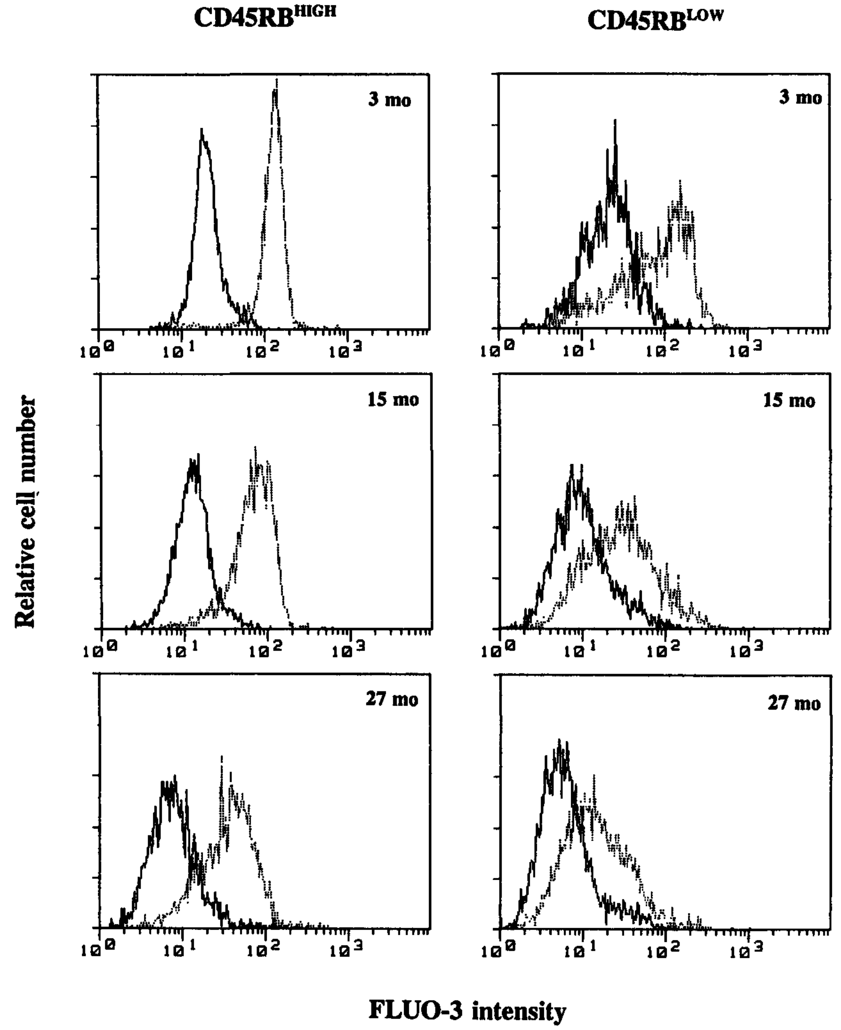

FIGURE 5. Relation between CD45RB expression and calcium mobilization. FLUO-3-labeled spleen cells from mice of different ages stained for CD4 and CD45RB were stimulated with ionomycin. Histograms of FLUO-3 intensity before stimulation (thick line) and at the peak of the response (thin line) are displayed. Results representative for three experiments are shown.

although an eventual inhibitory effect of the antibody would be expected to be higher on CD45RB$^{High}$ cells. Again, an additional decrease related to the age of the mice was noted in both subpopulations.

Altogether, it can be concluded that—regardless the age of the donor mice—CD4$^+$ T cells with a naive phenotype display a more profound calcium response to anti-CD3 or ionomycin than cells with a memory phenotype. In addition, CD4$^+$ T cells with a certain phenotype may still be heterogeneous in their stage of differentiation as can be concluded from differences in the capacity to increase intracellular calcium when such cells derived from mice of different ages are compared.

### DISCUSSION

Aging is accompanied by a decline in IL-2 production and in T-cell proliferation. Recently, we showed that the age-related decline in IL-2 production is accompanied by an increased production of IL-4 and IFN-$\gamma$ and that these events are related to an increase in the number of memory cells (Nagelkerken et al., 1991). Phenotypically, an age-related decrease in the fraction of CD45RB$^{High}$Pgp-1$^-$CD4$^+$ T cells in favor of CD45RB$^{Low}$Pgp-1$^{++}$CD4$^+$ T cells is observed (see Fig. 1). Pgp-1 has previously been associated with memory CD8$^+$ T cells (Budd et al., 1987; Butterfield et al., 1989) and this is consistent with the observation that the fraction of Pgp-1$^+$ T cells increases with age (Philosophe and Miller, 1989; Dobber et al., 1992; this paper). Moreover, Lee et al. (1990) demonstrated that secondary responses *in vivo* are associated with CD45RB$^-$CD4$^+$ T cells. Therefore, our observation in unmanipulated mice that the fraction of CD45RB$^{High}$Pgp-1$^-$CD4$^+$ T cells decreases with age in favor of CD45RB$^{Low}$Pgp-1$^{++}$CD4$^+$ T cells can be regarded as a shift from naive to memory cells. In the present study, we substantiated this observation by demonstrating that CD4$^+$ T cells from aged mice display a decreased expression of MEL-14, a marker recently also associated with naive CD4$^+$ T cells (Bradley et al., 1992). It is likely that an age-related increase in the fraction of memory T cells due to antigenic exposure combined with a decrease in the fraction of naive cells due to involution of the thymus and a limited life-span of these cells are the main reasons for the changed composition of the CD4$^+$ T-cell population. Interestingly, although CD8$^+$ T cells from aged mice display an increased expression of Pgp-1, these cells did not display a decreased expression of CD45RB or MEL-14 (data not shown). It has been suggested (Swain et al., 1991) that the differential expression of MEL-14 on CD4$^+$ T cells explains differences in terms of recirculation patterns between naive and memory cells, as has been demonstrated for sheep CD4$^+$ T cells (Mackay et al., 1990). Our data would then suggest that MEL-14 does not play a similar role in the case of CD8$^+$ T cells. Alternatively, despite the fact that splenic CD8$^+$ T cells from aged mice express increased levels of Pgp-1, CD8$^+$ T memory cells may be relatively absent from the spleen, a possibility we are currently investigating.

As indicated before, the age-related changes in the composition of the CD4$^+$ T-cell population is functionally reflected by a diminished production of IL-2. This phenomenon has been regarded as a defect caused by an inadequate calcium mobilization: indeed, it has been demonstrated that calcium mobilization declines during aging (Miller et al., 1987; Grossmann et al., 1989; this paper). On the basis of changes in the composition of the CD4$^+$ T-cell population, it is however, likely that the decreased ability to mobilize calcium is due to a change in the differentiation stage of the T cells and thus reflects a relative poor calcium response in memory cells. The fact that we observed no changes in calcium mobilization by B lymphocytes is not surprising because the generation of B lymphocytes is hardly affected by the aging process. This observation further showed that it is not likely that calcium mobilization in the cells of these mice is subject to a generalized suppression.

In the present study, we provide strong support for the idea that calcium mobilization plays a minor role in memory cells as compared to naive CD4$^+$ T cells. This conclusion is based on the assumption that CD45RB$^{High}$Pgp-1$^-$CD4$^+$ T cells represent the naive cells, and that CD45RB$^{Low}$Pgp-1$^{++}$CD4$^+$ T cells represent the memory cells. In view of the hypothesis that calcium mobilization is important for IL-2 gene expression, our present observation that CD45RB$^{Low}$Pgp-1$^{++}$CD4$^+$ memory T cells do not mobilize calcium as efficient as CD45RB$^{High}$Pgp-

1⁻CD4⁺ naive T cells is also consistent with difference in IL-2 production by these cell populations (Bottomly et al., 1989; Lee et al., 1990; Nagelkerken et al., 1991). In these studies, we showed that in response to Con A, $CD45RB^{Low}CD4^+$ T cells produced 15 fold lower levels of IL-2 as compared to $CD45RB^{High}CD4^+$ T cells. Moreover, we observed that $CD45RB^{High}CD4^+$ T cells from young mice produced substantially more IL-2 than their old counterparts, which again is consistent with our present observation that naive $CD4^+$ T cells from 3-month-old mice increase intracellular calcium more efficiently than the naive $CD4^+$ T cells derived from 15-month-old or 27-month-old animals. Furthermore, this observation showed that $CD4^+$ T cells with a naive phenotype may still be different with regard to their stage of differentiation.

The fact that in our previous studies (Nagelkerken et al., 1991) IL-2 production could be enhanced by stimulating $CD45RB^{Low}CD4^+$ T cells with the combination of PMA and ionomycin is in line with our present observation that ionomycin—in contrast to anti-CD3—was capable to induce an increase in intracellular calcium (Table 1). That ionomycin did not induce calcium mobilization in all old $CD4^+$ T cells was a consistent finding that may be due to the increased heterogeneity in the differentiation stage of these cells and that is possibly due to an increased activity of calcium pumps.

Our results suggest that during the process of differentiation from naive to memory cells, $CD4^+$ T cells lose their capacity to increase intracellular calcium when stimulated in a physiological manner. A possible explanation might be that cAMP levels are increased in memory cells, which, in turn, is inhibitory for the hydrolysis of phosphatidylinositol and thus calcium mobilization (Friedrich et al., 1989). We are currently investigating this possibility, which is supported by our observation that $CD4^+$ T-cell populations enriched for memory cells are less sensitive for inhibition by cAMP inducers (Dobber and Nagelkerken, manuscript in preparation). Alternatively, memory cells may depend on other intracellular pathways that are mediated, for example, by CD28 (Fraser et al., 1991). Interestingly, anti-CD28 can induce high levels of IL-2 in human $CD4^+$ T memory cells· (de Jong et al., 1991). Because memory $CD4^+$ T cells are poor responders with regard to calcium mobilization, it is likely that alternative intracellular pathways play a role in the induction of lymphokines that are more specific for these cells.

### MATERIALS AND METHODS

### Mice

Male CBA/Rij mice of three different ages (3, 15, and 27 months old) were derived from the SPF cohorts of the animal facilities of ITRI-TNO (Rijswijk, The Netherlands). In all experiments, pools of cells derived from four mice were used.

### Cells

$CD4^+$ T cells were isolated as described previously (Nagelkerken et al., 1991) by depletion of $CD8^+$ T cells and macrophages using anti-Ly-2, anti-Mac-1$\alpha$, and anti-I-$A^{b,d,q}$. Rather than depleting the cells by indirect panning, depletion was achieved with Magnisort-G (Dupont, Wilmington, DE), saturated with affinity-purified goat-anti-rat-Ig. Simultaneously, B cells were depleted with Magnisort-M (Dupont), according to the instructions of the manufacturer. Cells adherent to the magnetic particles were removed with a magnet (Dynal AS, Oslo, Norway). To achieve a purity of 95% $CD4^+$ T cells, the nonadherent fraction was next incubated with anti-asialo-GM-1 (WAKO Chemicals, Neuss, Germany) and NK cells were removed with Magnetic goat-anti-rabbit IgG (Collaborative Research, Bedford, MA). B cells were purified by treatment of spleen cells (after lysis of the erythrocytes) with anti-Thy-1.2 (Cedarlane, Hornby, Canada) and rabbit complement.

### Phenotypic Analysis

The purity of the various cell populations was verified by flow cytometry using a FACScan (Becton Dickinson, Sunnyvale, CA). Changes in the composition of the $CD4^+$ T-cell population were studied in triple staining experiments using unseparated spleen cells. Pooled spleen cells from four mice were depleted of erythrocytes and incubated (30 min on ice) with anti-CD45RB (antibody 23G2; dilution 1/200; Texstar, Dallas, Texas), washed and incubated with FITC-labeled mouse-anti-rat-IgG (dilution:1/200; Jackson

Immunoresearch Laboratories, West Grove, PA). After washing the cells, these were simultaneously incubated with PE-conjugated anti-Pgp-1 (dilution: 1/25; Pharmingen, San Diego, CA) and anti-CD4$^{Red613}$ (dilution 1:100; GIBCO, Gaithersburg, MD) in the presence of 10% normal rat serum to prevent binding to already bound anti-rat IgG. CD4$^+$ T cells were gated on the basis of forward scatter and fluorescence intensity in order to exclude macrophages from the analysis. Gated cells were analyzed for CD45RB (green) and Pgp-1 (red) expression, simultaneously. The expression of MEL-14 (Gallatin et al., 1983) on CD4$^+$ or CD8$^+$ T cells (stained with the appropriate PE-conjugated antibodies, Becton Dickinson) was studied using hybridoma supernatant in combination with FITC-conjugated mouse-anti-rat Ig. Background staining by isotype controls or by a second antibody alone resulted in a peak fluorescence ranging from 1 to $5 \times 10^0$ on the used log scales with a mean fluorescence intensity of 2.

### Calcium Mobilization

B cells or CD4$^+$ T cells were labeled with INDO-1-AM (Molecular Probes, Eugene, OR), as described by Rabinovitch et al. (1986) and analysed for their ability to mobilize calcium after stimulation with the use of a FACS-analyzer equipped with an ARC-lamp (Becton Dickinson), as described by Grifioen et al. (1989). Cells were excited at 362 nm, wheras emission was studied at 405 and 485 nm. Data were collected and processed using INCA software, as described elsewhere (Keij et al., 1989).

T cells were incubated (30 min on ice) with 10 $\mu$ g/ml anti-CD3 antibody 145-2C11 (Leo et al., 1987), washed, resuspended in RPMI 1640 containing 5% FCS, and kept on ice until assay. A sample of these cells was then resuspended in prewarmed (37°C) medium to a concentration of $10^5$/ml and immediately analyzed after the addition of goat-anti-rat-Ig cross-reactive with hamster Ig (NORDIC, Tilburg, The Netherlands; final dilution 1/40). Fifty thousand events were collected over a period of about 5 min. B cells were stimulated with affinity-purified goat-anti-mouse Ig at a concentration of 100 $\mu$g/ml. As an alternative to anti-CD3 or anti-Ig, cells were stimulated with 1 $\mu$M ionomycin in order to establish the maximal ratio free INDO-1/calcium-bound INDO-1. The minimal ratio was determined by using the same approach in the presence of 8 mM EGTA. Responding cells are those cells in which the ratio exceeded the ratio in unstimulated cells+3 SD.

To study calcium mobilization in relation to phenotype, an alternative procedure was used. Spleen cells ($10^7$/ml) were labeled with 1 $\mu$M Fluo-3 (Molecular Probes, Eugene, OR), washed, and simultaneously incubated with anti-CD4$^{Red613}$ and anti-Pgp-1-PE as described before. After incubation, the cells were washed and kept on ice until assay. Alternatively, Fluo-3-labeled cells were incubated with 23G2, washed, incubated with PE-labeled rabbit $(Fab)_2$-anti-rat-Ig (dilution, 1/50; Serotec, Oxford, England). After a washing step, the cells were incubated with anti-CD4$^{Red613}$ in the presence of 10% normal rat serum. Cells were stimulated with anti-CD3 or ionomycin as described for INDO-1.

Calcium mobilization (green fluorescence) was measured on a FACScan using a live gate around the CD4$^+$ T cells, and recorded in relation to the phenotype of the cells (red fluorescence). Data were analyzed in CONSORT 30 after creating a simulated time scale employing the CONVERT program (Becton Dickinson).

### ACKNOWLEDGMENTS

We are grateful to Jan Rozing, Angelika Dräger, and Ruud Dobber for helpful discussion and critically reviewing the manuscript, and to Marga van Setten for helpful technical assistance.

*(Received April 2, 1992)*

*(Accepted June 23, 1992)*

### REFERENCES

Bottomly K., Luqman M., Greenbaum L., Carding S., West J., Pasqualini T., and Murphy D.B. (1989). A monoclonal antibody to murine CD45R distinguishes CD4 T cell populations that produce different cytokines. Eur. J. Immunol. **19:** 617–623.

Bradley L.M., Atkins G.S., and Swain S.L. (1992). Long-term CD4$^+$ memory T cells from the spleen lack MEL-14, the lymph node homing receptor. J. Immunol. **148:** 324–331.

Budd R.C., Cerottini J.-C., Horvath C., Bron C., Pedrazzini T., Howe R.C., and MacDonald H.R. (1987). Distinction of virgin and memory T lymphocytes. Stable acquisition of the Pgp-1 glycoprotein concomitant with antigenic stimulation. J. Immunol. **138:** 3120–3129.

Butterfield K., Fathman C.G., and Budd R.C. (1989). A subset of memory CD4$^+$ helper T lymphocytes identified by expression of Pgp-1. J. Exp. Med. **169:** 1461–1466.

Crabtree G.R. (1989). Contingent genetic regulatory events in T lymphocyte activation. Science **243:** 355–361.

de Jong R., Brouwer M., Miedema F., and Van Lier R.A.W. (1991). Human CD8$^+$ T lymphocytes can be divided into CD45RA$^+$ and CD45RO$^+$ cells with different requirements for activation and differentiation. J. Immunol. **146:** 2088–2094.

Dobber R., Hertogh-Huijbregts A., Rozing J., Bottomly K., and Nagelkerken L. (1992). The involvement of the intestinal microflora in the expansion of CD4$^+$ T cells with a naive phenotype in the periphery. Dev. Immunol. **2:** 141–150.

Ernst D.B., Hobbs M.V., Torbett B.E., Glasebrook A.L., Rehse M.A., Bottomly K., Hyakawa K., Hardy R.R., and Weigle W.O. (1990). Differences in the expression profiles of CD45RB, Pgp-1, and 3G11 membrane antigens and in the patterns of lymphokine secretion by splenic CD4$^+$ T cells from young and aged mice. J. Immunol. **145:** 1295–1302.

Fraser J.D., Irving B.A., Crabtree G.R., and Weiss A. (1991). Regulation of interleukin-2 gene enhancer activity by the T cell accessory molecule CD28. Science **251:** 313–316.

Friedrich B., Cantrell D.A., and Gullberg M. (1989). Increased cyclic AMP levels block interleukin-2-induced protein kinase C substrate phosphorylation but not the mitogenic response. Eur. J. Immunol. **19:** 1111–1116.

Gallatin W.M., Weissman I.L., and Butcher E.C. (1983). A cell-surface molecule involved in organ-specific homing of lymphocytes. Nature **304:** 30–34.

Gillis S., Kozak R., Durante M., and Weksler M.E. (1981). Immunological studies of aging. Decreased production of and response to T cell growth factor by lymphocytes from aged humans. J. Clin. Invest. **67:** 937–942.

Griffioen A.W., Rijkers G.T., Keij J., and Zegers B.J.M. (1989). Measurement of cytoplasmic calcium in lymphocytes using flow cytometry. Kinetic studies and single cell analysis. J. Immunol. Meth. **120:** 23–27.

Grossmann A., Ledbetter J.A., and Rabinovitch P.S. (1989). Reduced proliferation in T-lymphocytes in aged humans is predominantly in the CD8$^+$ subset, and is unrelated to defects in transmembrane signaling which are predominantly in the CD4$^+$ subset. Exp. Cell Res. **180:** 367–382.

Hertogh-Huijbregts A., Vissinga C., Rozing J., and Nagelkerken L. (1990). Impairment of CD3-dependent and CD3-independent activation pathways in CD4+ and in CD8+ T cells from old CBA/Rij mice. Mech. Ageing Dev. **53:** 141–155.

Hivroz-Burgaud C., Clipstone N.A., and Cantrell D.A. (1991). Signaling requirements of the expression of the transactivating factor NF-AT in human T lymphocytes. Eur. J. Immunol. **21:** 2811–2819.

Keij J.F., Griffioen A.W., The Th., and Rijkers G.T. (1989). INCA: Software for consort 30 analysis of flow cytometric calcium determinants. Cytometry **10:** 814–817.

Lee W.T., Yin X.-M., and Vitetta E.S. (1990). Functional and ontogenetic analysis of murine CD45R$^{hi}$ and CD45R$^{lo}$CD4$^+$ T cells. J. Immunol. **144:** 3288–3295.

Leo O., Foo M., Sachs D.H., Samelson L.E., and Bluestone J.A. (1987). Identification of a monoclonal antibody specific for a murine T3 polypeptide. Proc. Natl. Acad. Sci. USA **84:** 1374–1378.

Lerner A., Yamada T., and Miller R.A. (1989). Pgp-1$^{hi}$ T lymphocytes accumulate with age in mice and respond poorly to concanavalin A. Eur. J. Immunol. **19:** 977–982.

Mackay C.R., Marston W.L., and Dudner L. (1990). Naive and memory T cells show distinct pathways of lymphocyte recirculation. J. Exp. Med. **171:** 801–817.

Miller R.A., Jacobson B., Weil G., and Simons E.R. (1987). Diminished calcium influx in lectin-stimulated T cells from old mice. J. Cell. Physiol. **132:** 337–342.

Nagelkerken L., Hertogh-Huijbregts A., Dobber R., and Dräger A. (1991). Age-related changes in lymphokine production related to a decreased number of CD45RB$^{hi}$CD4$^+$ T cells. Eur. J. Immunol. **21:** 273–281.

Philosophe B., and Miller R.A. (1989). T lymphocyte heterogeneity in old and young mice: Functional defects in T cell selected for poor calcium signal generation. Eur. J. Immunol. **19:** 695–699.

Rabinovitch P.S., June C.H., Grossmann A., and Ledbetter J.A. (1986). Heterogeneity among T cells in intracellular free calcium responses after mitogen stimulation with PHA or anti-CD3. Simultaneous use of INDO-1 and immunofluorescence with flow cytometry. J. Immunol. **137:** 952–961.

Shaw J.-P., Utz P.J., Durand D.B., Toole J.J., Emmel E.A., and Crabtree G.R. (1988). Identification of a putative regulator of early T cell activation genes. Science **241:** 202–205.

Swain S.L., Bradley L.M., Croft M., Tonkonogy S., Atkins G., Weinberg A.D., Duncan D.D., Hedrick S.M., Dutton R.W., and Huston G. (1991). Helper T-cell subsets: phenotype, function and the role of lymphokines in regulating their development. Immunol. Rev. **123:** 115–144.

Thoman M.L., and Weigle W.O. (1981). Lymphokines and aging: Interleukin-2 production and activity in aged animals. J. Immunol. **127:** 2102–2106.

Verweij C.L., Guidos C., and Crabtree G.R. (1990). Cell type specificity and activation requirements for NFAT-1 (Nuclear Factor of Activated T-cells) transcriptional activity determined by a new method using transgenic mice to assay transcriptional activity of an individual nuclear factor. J. Biol. Chem. **265:** 15788–15795.

